# Let-7e sensitizes epithelial ovarian cancer to cisplatin through repressing DNA double strand break repair

**DOI:** 10.1186/s13048-017-0321-8

**Published:** 2017-04-04

**Authors:** Man Xiao, Jing Cai, Liqiong Cai, Jinghui Jia, Lisha Xie, Ying Zhu, Bangxing Huang, Dongdong Jin, Zehua Wang

**Affiliations:** 1 Department of Obstetrics and Gynecology, Union Hospital, Tongji Medical College, Huazhong University of Science and Technology, No. 1277 Jiefang Avenue, Wuhan, 430022 China; 2 Department of Obstetrics and Gynecology, Air Force General Hospital, PLA, Beijing, 100142 China; 3 Department of Pathology, Union Hospital, Tongji Medical College, Huazhong University of Science and Technology, No. 1277 Jiefang Avenue, Wuhan, 430022 China

**Keywords:** Let-7e, Ovarian cancer, Cisplatin, DSB, BRCA1, Rad51

## Abstract

**Background:**

Resistance to platinum-based chemotherapy remains a great challenge for ovarian cancer treatment. The human let-7 family contains 13 members located on nine different chromosomes, and most members have been implicated in the modulation of drug sensitivity in cancers. Our previous study showed that deregulation of let-7e in epithelial ovarian cancer (EOC) promoted the development of resistance to cisplatin. In the present study, we aimed to investigate the underlying mechanism and further evaluate the clinical value of let-7e in predicting chemo-response and prognosis in EOC.

**Results:**

In situ hybridization assays revealed a significantly decreased expression of let-7e in chemo-resistant EOC tissues compared with chemo-sensitive cases. Transfection with let-7e agomir sensitized EOC cells to cisplatin, down-regulated BRCA1 and Rad51 expression, and repressed the repair of cisplatin-induced DNA double strand break, while let-7e inhibitor exerted the opposite effects. In human EOC tissues, BRCA1 and Rad51 levels were increased in the chemo-resistant group compared with the sensitive group and were negatively correlated with let-7e. Low let-7e and high Rad51 were significantly associated with poor progression-free survival and overall survival and multivariate regression analyses showed that let-7e was an independent predictor for overall survival and chemotherapy response in EOC. Receiver operating characteristic analysis indicated that let-7e level was highly predictive of resistance to platinum-taxane chemotherapy with an area under the curve of 0.826.

**Conclusions:**

In EOC, low let-7e leads to activation of BRCA1 and Rad51 expression and subsequent enhancement of DSB repair, which in turn results in cisplatin-resistance. Let-7e is a potential predictor for survival and chemo-response in EOC and re-expression of let-7e might be an effective strategy for overcoming chemo-resistance.

**Electronic supplementary material:**

The online version of this article (doi:10.1186/s13048-017-0321-8) contains supplementary material, which is available to authorized users.

## Background

Ovarian cancer remains one of the leading causes of gynecological-cancer-associated death [1]. Late diagnosis and chemotherapy resistance account for the stubbornly high mortality associated with ovarian cancer. Platinum drugs are the most widely used first-line chemotherapy agents for treatment of epithelial ovarian cancer (EOC), which exert cytotoxic effect through reacting with DNA and subsequently inducing intra- or inter-strand cross-link or single nucleotide damage. Inter-strand cross-link can lead to double strand break (DSB) that represents a form of lethal DNA damage and can be repaired through homologous recombination (HR) and non-homologous end-joining (NHEJ) [2]. HR is thought to be a conserved and error-free pathway to repair DSB compared with NHEJ, which is regarded as imprecise and potentially mutagenic [2, 3]. In addition, HR is related with cisplatin sensitivity in several solid tumors such as esophageal squamous carcinoma and embryonal carcinoma [4, 5]. The inhibition of HR repair capacity could effectively sensitize ovarian cancer cells to cisplatin [6]. However, the regulatory mechanism for HR repair of cisplatin-induced DSB is largely unknown.

MicroRNAs (miRNAs) represent a class of non-coding RNAs (~22 nt) with regulatory functions through complete or incomplete base paring with the 3’ untranslated region (UTR) of target genes, leading to RNA degradation or translation repression [7]. To date, enormous evidence has highlighted the implication of miRNAs in chemo-resistant phenotype of cancers. Down-regulation of miR-186, miR-203, miR-181b or miR-497 could influence the sensitivity of ovarian, breast, lung or gastric cancer to cisplatin treatment [8, 9]. As the second discovered miRNA after lin-4, let-7 was first identified in 2000, and its 13 family members share sequence homology in seed regions. Emerging data suggest that let-7 family members display different activities, and most members have been implicated in the modulation of drug sensitivity in cancers [10]. Our previous study indicated that over-expression of let-7e increased the sensitivity to cisplatin in EOC cells in vitro and in vivo [11]. Nevertheless, the underlying mechanism remains unclear. The DNA damage induced by ionizing radiation significantly altered the expression of let-7e [12, 13], and let-7e was identified as one of the miRNAs that influenced γH2AX foci formation [14], which is a surrogate marker of DSB [15]. We therefore hypothesized that let-7e regulates the response to cisplatin in EOC through impacting the repair of cisplatin-induced DSB. In this study, we provided evidence supporting the involvement of let-7e in DSB repair and cisplatin-resistance and explored the prognostic values of let-7e and two key factors implicated in HR repair, BRCA1 and Rad51, for survival and chemo-response in EOC.

## Methods

### Patients and tissue samples

A total of 84 tissue samples obtained from EOC patients (median age, 52 years; range, 32–72 years) who underwent primary surgery and adjuvant platinum-based chemotherapy at Wuhan Union Hospital between August 2008 and October 2015 were studied. All patients received radical surgery followed by standard platinum-taxane chemotherapy. Chemo-resistant tumors were defined as those with relapse within six months after completing chemotherapy or progress during the primary chemotherapy [16]. The relapses were diagnosed based on clinical symptoms, radiological evidence or rising CA125 [16]. The medical records of patients were reviewed to collect data regarding clinicopathological characteristics and treatment. The following parameters were recorded: age, histological subtypes, International Federation of Gynecology and Obstetrics (FIGO) stage, tumor categories and chemotherapy response. In addition, the follow-up data (median, 25 months; range, 4–59 months) were collected. Overall survival (OS) was defined as the time from initial resection to death or the last follow-up date; profession-free survival (PFS) was defined as the time from surgery to relapse or the last follow-up date. The diagnosis was pathologically confirmed in all patients and the patients who underwent chemotherapy or radiotherapy prior to surgery were excluded.

Formalin-fixed and paraffin embedded (FFPE) tissues were used for in situ hybridization (ISH) and immunohistochemistry (IHC). H&E staining was performed for each paraffin block to exclude those noncancerous blocks. The use of specimens and archiving of patient data were approved by the ethical committee of Union Hospital.

### ISH

The detection of let-7e expression in FFPE ovarian cancer tissues was performed according to the robust one-day ISH protocol based on the use of double digoxigenin (DIG)-labeled Locked Nucleic Acids (LNA) probes (Exiqon, Denmark), which retain a larger difference in melting temperature between a complementary target and a highly similar target sequences and have the ability to discriminate highly similar miRNA family members at the optimized hybridization temperature, like the let-7 family members [17]. Only those tissues with positive U6 expression were admitted for subsequent let-7e detection to exclude false-negative results. In short, after deparaffinization in xylene and graded ethanol dilution, six-micrometer sections were treated with 15 μg/ml proteinase-K at 37 °C for 7 min and dehydrated with ethanol. The sections were hybridized with 1 nM U6 snRNA and 20 nM let-7e or a scramble control probe at 60 °C for 1 h. Coverslips were sealed with Fixogum Rubber Cement during hybridization. A strict wash was then performed in pre-heated standard saline citrate (SSC) buffers at 60 °C for 5 min each: once in 5 × SSC, twice in 1 × SSC, twice in 0.2 × SSC and once in 0.2 × SSC at room temperature. The samples were rinsed with PBS containing 0.1% Tween-20 (PBST) solutions and blocked in blocking solution (2% sheep serum, 1% bovine serum albumin in PBST) for 15 min at room temperature. Thereafter, the samples were incubated with an alkaline phosphatase (AP)-conjugated anti-DIG antibody (1:1600 dilution; Roche, Mannheim, Germany) at room temperature for 60 min. After washing twice in PBST, the sections were incubated with NBT/BCIP (Roche) complemented with 0.2 mM Levamisole Hydrochloride (TGI, Tokyo, Japan) at 30 °C for 2 h to develop the dark-blue NBT-formazan precipitate. Slides were then washed twice in KTBT buffer and counterstained with nuclear fast red (Vector Laboratories, CA, USA).

### IHC

Sections (4 μm thick) were dewaxed in xylene and rehydrated through descending ethanol series to water. Antigen retrieval was performed in citrate buffer (PH 6.0) through microwave irradiation. After endogenous peroxidase being blocked with 3% hydrogen peroxide, slides were incubated with polyclonal rabbit anti-BRCA1 (1:100 dilution; Proteintech Group, Chicago, USA) or anti-Rad51 polyclonal antibody (1:100 dilution; Santa Cruz, Texas, USA) at 4 °C overnight and then treated with a biotinylated secondary antibody at room temperature for 30 min. After that, further incubation with horseradish peroxidase labeled avidin, DAB color development and counterstaining with hematoxylin were performed. Negative controls were accomplished by replacing the primary antibody with PBS.

### Scoring of ISH and IHC

The expression of let-7e, BRCA1 and Rad51 were evaluated independently by two pathologists blinded to the patient data of the samples. In case of discrepancy, the slide was reexamined by the third pathologist. In each slide, the proportion and intensity of positive staining of tumor cells were evaluated in five randomly selected high-power fields and a staining score (0–9) was determined by multiplying the positive proportion score (0, 0%; 1, < 25%; 2, 25–50%; 3, > 50%) with the staining intensity score (0, negative; 1, light blue or yellow; 2, blue or yellow; 3, dark blue or yellow). The median scores were used as the cutoff values to define the high and low expression of let-7e, BRCA1 and Rad51.

### Cell lines and cell culture

The A2780, HO8910, ES2, CAOV3 and SKOV_3_ human epithelial ovarian cancer cell lines were obtained from China Center for Type Culture Collection (CCTCC). The cisplatin-sensitive cell line OV2008 and its resistant variant C13K were initially obtained from Dr. Rakesh Goel of the Ottawa Regional Cancer Center, Canada and stored in our laboratory. Cells were cultured in Dulbecco’s Modified Eagle Medium (DMEM) supplemented with 10% fetal bovine serum under a humidified atmosphere containing 5% CO_2_ at 37 °C.

### Transfections

The let-7e agomir and inhibitor were synthesized by Genepharma (Shanghai, China). As the cholesterol-conjugated 2’-O-methyl-modified miRNA mimics, miRNA agomir has a greater affinity for cell membrane and can function stronger and longer. MiRNA inhibitors are 2’-O-methyl-modified oligoribonucleotides with the ability to effectively and sequence-specifically block RISC-associated miRNP (miRNA-containing ribonucleic protein) activity. Twenty-four hours before transfection, cells were seeded in a six-well plate at a concentration of 4 × 10^5^ cells per well. Upon reaching 60–70% confluence, cells were transfected with 30 nM let-7e agomirs, 200 nM let-7e inhibitors, or corresponding negative control using PEI according to the manufacture^’^s instructions. The transfection efficiency was verified by qRT-PCR.

### MTT assay

After transfection, a density of 5000 cells per well were seeded onto 96-well plates and exposed to a series of cisplatin for 48 h. MTT reagent (at a final concentration of 10%; Sigma, USA) was then added to each well and further incubated for 4 h at 37 °C before 100 μl lysis buffer (10 g of SDS, 5 ml isopropanol and 1 ml 1 mM hydrochloric acid) was added to dissolve the MTT formazan product. Absorbance at 570 nm was measured with a microplate reader (Bio-Rad, Hercules, CA, USA) and cellular survival fractions were calculated by normalizing the optical density of cisplatin treated wells to that of untreated controls.

### Colony-formation assay

Approximately five hundred transfected cells per well were plated into six-well plates and incubated with cisplatin for 48 h and then allowed to recover for two weeks. Colonies derived from surviving cells were stained with 0.1% crystal violet and those more than 50 cells per colony were counted manually.

### Real-time PCR for let-7e

Total RNA of cultured cells were extracted using Trizol (Takara, Japan) and their quality and quantity were checked by Nanodrop 2000 (Thermo Scientific, Waltham, MA, USA) before the synthesis of cDNA using the RevertAid First Strand complementary DNA Synthesis Kit (Thermo Scientific). Bulge-Loop™ hsa-let-7e-5p qRT-PCR Primer Set (RiboBio, Guangzhou, China) was used to analyze the expression of let-7e according to the manufacturer^’^s instructions. The universally expressed U6 snRNA (RiboBio) was used for let-7e normalization. PCR reactions were carried out on an ABI PRISM 7300 Step-One Plus instrument (Applied Biosystems, Foster City, CA, USA) with SYBR Green PCR Master Mix (Takara). The cycling method for the reverse transcription reaction was as follows: 70 °C for 10 min, 2 min on ice followed by 42 °C for 1 h and 70 °C for 10 min. The cycling conditions of PCR consist of an initial denaturizing step of 20s at 95 °C and 40 cycles of denaturing at 95 °C for 10s, annealing at 60 °C for 20s and extension at 70 °C for 10s.

### Real-time PCR for mRNA expression

After total RNA was isolated from cells, the cDNA was synthesized based on the universal primers using a reverse transcription kit (Takara). The sequences of PCR primers were displayed in Additional file 1: Table S1. β-actin was used as internal control. The PCR profile was 95 °C for 1 min, and then followed by 40 cycles of 95 °C for 15 s, 60 °C for 1 min. Each reaction was performed in triplicate.

### Single-cell gel electrophoresis (Comet assay)

To evaluate the repair capacity of DSB caused by cisplatin, approximately 50000 ovarian cancer cells per well were seeded into 12-well plates and treated with low concentration of cisplatin (3 μM for A2780, 5 μM for OV2008) for 8 h. After incubation, cultures were allowed to recover for 0, 4, 8 and 24 h. Thereafter, cells were trypsinized and harvested. Approximately 2 × 10^4^ cells per aliquot were suspended with 20 μl PBS.

To detect DSB, neutral comet assays (pH 8.5) were performed as described by Wojewodzka M et al. [18] with slight modification. In brief, fully frosted microscope slides were covered with 1% normal melting point agarose (the first layer) and 0.5% low melting point agarose (LMP, the second layer) sequentially. Then, 100 μl of the mixture of 0.7% LMP agarose and cell suspension (the third layer) was pipetted onto the center of the second agarose layer. At each step, sides were placed on ice for 10–15 min to allow the agarose to solidify and maintain cooling. After that, slides were immersed into ice-cold lysis solution (2.5 M NaCl, 100 mM Na_2_EDTA, 10 mM Tris–HCl and 1% Triton X-100) at 4 °C for 1.5 h. The slides were then directly placed in neutral electrophoresis buffer (300 mM sodium acetate, 100 mM Tris–HCl, pH 8.5) and left for 30 min before being electrophoresed at 16 V and 50 mA (0.4 V/cm) for 1 h on ice. After that, slides were stained with 10 μl of 2 μg/ml ethidium bromide and visualized with a microscope at a 4× objective.

Comet Assay Software Project (CASP) was used for image analysis. Tail moment (TM, the length of the comet tail multiplied by the intensity of fluorescence in the tail) [19] was used to quantify the DNA damage. The residual DNA damage of cells over a recovery period of 4, 8 and 24 h was normalized to that of freshly harvested cells after incubation with cisplatin. One hundred randomly selected cells for each group were analyzed in three separate experiments.

### Detection of γ-H2AX foci by immunofluorescence

In response to DSB, the histone H2AX is phosphorylated by ataxia telangiectasia mutated (ATM) and ataxia Rad3-related (ATR) kinases and recruited to the site of damage along with an extensive network of proteins. In general, γ-H2AX foci (clusters of phosphorylated H2AX) has been widely applied as a surrogate marker of DSB after cellular exposure to ionizing radiation (IR) or drugs [15]. After transfection, cells were grown on coverslips in a 24-well plate (20, 000 cells/well) and treated with cisplatin (3 μM for A2780, 5 μM for SKOV_3_) for 8 h. Then cells were allowed to recover for another 8 h before fixed with 4% paraformaldehyde. After washing with PBS, cells were permeabilized with 0.5% Triton X-100 for 30 min, blocked with 3% bovine serum albumin (BSA) containing 0.1% Tween-20 for 1 h and immunostained with antibody against γ-H2AX (1:400 dilution; Cell Signaling Technology, Danvers, MA, USA) overnight at 4 °C, followed by incubation with Cy3-labled antibody to Rabbit IgG (H + L) (1:1000 dilution; KPL, Gaithersburg, MD, USA) at room temperature for 1 h. Then nuclei were counterstained with DAPI (100 ng/ml; Roche). Coverslips with cells were sealed before acquiring images with an A1-Si Nikon confocal laser scanning microscope. At least 100 cells per experiment were evaluated and cells with five or more foci were defined as positive. Results were expressed as the percentage of cells with positive γ-H2AX foci from three independently replicated experiments.

### Western blotting

Proteins from whole cell lysates were separated by 10% SDS-PAGE gel and transferred to a polyvinylidene fluoride membrane (Bio-Rad, Hercules, CA, USA). The membrane was then blocked and incubated with rabbit anti-BRCA1 (1:600 dilution; Proteintech Group, Chicago, USA), rabbit anti-Rad51 (1:750 dilution; Santa Cruz, Texas, USA) or mouse anti β-actin (1:4000 dilution; Proteintech Group) at 4 °C overnight. Peroxidase-labeled anti-rabbit or anti-mouse secondary antibodies (1:5000 dilution; KPL, Gaithersburg, MD, USA) were used to detect the specific protein-antibody complex. Bands were visualized by an ECL substrate (Bio-Rad) and scanned using Image Lab Software in Molecular Imager® ChemiDoc^TM^ XRS+ (Bio-Rad). Equal protein loading was verified by β-actin signal.

### Statistical analysis

All statistical analyses were carried out with the SPSS 20.0 software (SPSS). Student^’^s *t*-test or Mann Whitney test were used for comparisons between groups as appropriate. The relationship between the expression of let-7e, BRCA1 and Rad51 and EOC clinicopathological characteristics was analyzed by Fisher exact test. Survival curves were estimated by Kaplan-Meier method and compared with log-rank test. The risk for patient survival and chemotherapy response were assessed by Cox and logistic regression analyses, respectively. Receiver operating characteristic (ROC) curve analyses were performed to evaluate the capacity of let-7e, BRCA1 and Rad51 to predict chemotherapy response of EOC. P values less than 0.05 were considered statistically significant.

## Results

### Let-7e is correlated with chemo-resistance in epithelia ovarian cancer

**Fig. 1:**
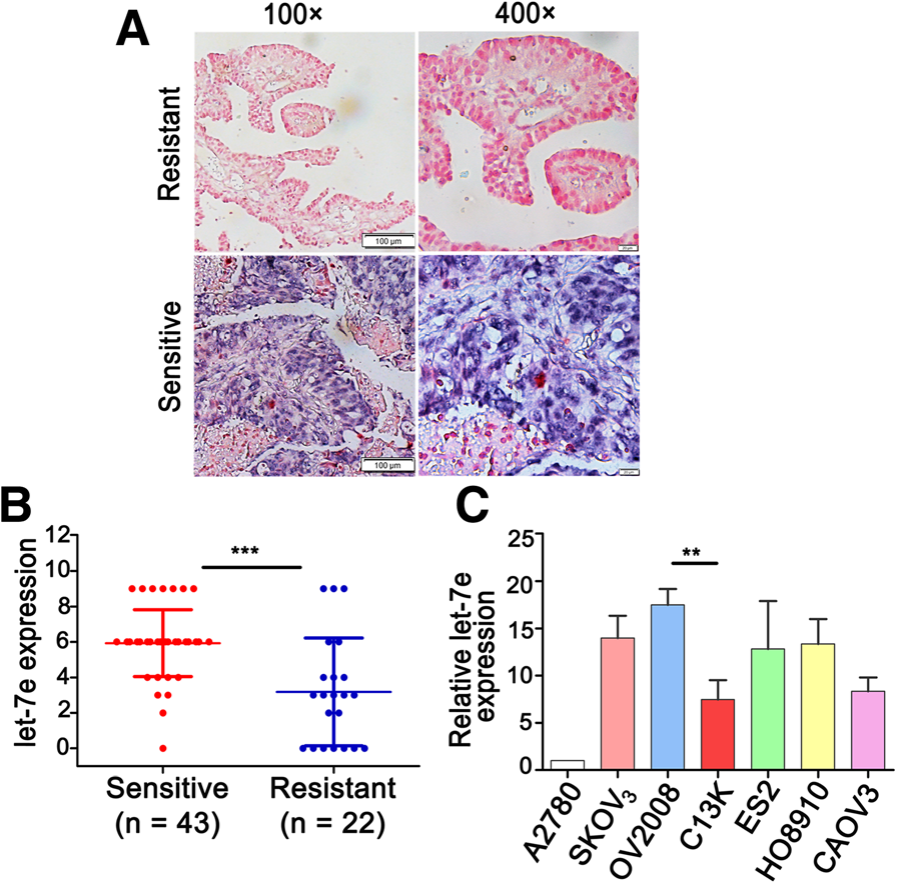
Let-7e expression is significantly decreased in chemo-resistant epithelial ovarian cancer (EOC). **a** Representative images of let-7e via in situ hybridization in one of the chemo-resistant (upper) versus chemo-sensitive (down) ovarian cancer specimens. The positive let-7e LNA-ISH signal is blue. **b** The corresponding histogram of the semi-quantification of let-7e level in two groups. Mann Whitney test, ***P < 0.0001. **c** Validation of let-7e using qRT-PCR in seven ovarian cancer cell lines. C13K is the corresponding cisplatin-resistant variants of ovarian cancer cell OV2008. The let-7e level in A2780 were set as 1. Student’s *t*-test, **P < 0.01

To determine whether let-7e plays a role in the generation of chemotherapy resistance in EOC, we compared let-7e expression in chemo-resistant EOC (*n* = 22) versus chemo-sensitive EOC (*n* = 43) using ISH. The results showed a significantly decreased let-7e expression in the chemo-resistant EOC (Fig. 1a and b). Next, let-7e expression was assessed in seven ovarian cancer cell lines. Let-7e was detectable in all seven cell lines with the lowest level in A2780 and the highest level in OV2008. Moreover, the let-7e expression was significantly higher in OV2008 compared with its paired resistant line C13K (Fig. 1c). These results suggest that the decreased expression of let-7e may be involved in the development of resistance to platinum-based chemotherapy in EOC.

**Fig. 2:**
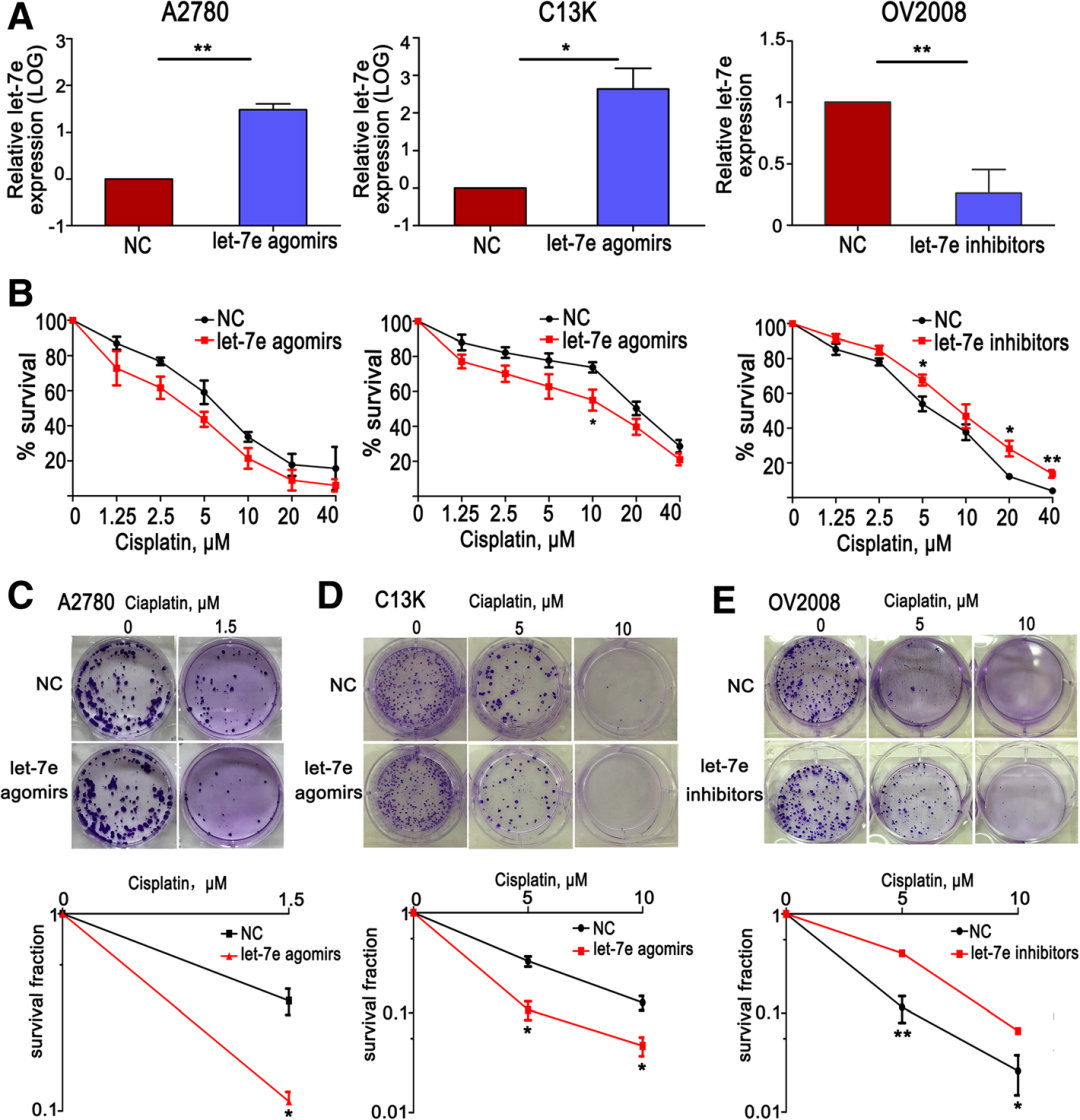
Overexpression of let-7e increases the sensitivity of ovarian cancer cell lines to cisplatin. **a** qRT-PCR analyses of let-7e expression in A2780, C13K and OV2008 after transfection with let-7e agomirs or inhibitors. **b** Cell viability was assessed by MTT assay after incubation with a series of cisplatin. **c**, **d** and **e** Clonogenic cell-survival assay analysis of cell viability after transfected with let-7e agomirs or inhibitors and treated with cisplatin. Representative images of colonies formed by A28780, C13K and OV2008 cell lines and the corresponding statistic graphs are shown. Student’s *t*-test, *P < 0.05, **P < 0.01

We next examined the effect of let-7e on cytotoxicity of cisplatin. The let-7e expression was manipulated by agomir and inhibitor (Fig. 2a). Since miRNA inhibitor technologies rely on competitively inhibited the effect of miRNA on their target genes, we detected the influence of let-7e inhibitor on the mRNA levels of RFX6 (regulatory factor X 6) [20], caspase 3 (CASP3) [21], matrix metalloproteinase-9 (MMP9) [22] and enhancer of zeste 2 (EZH2) [23], which have been validated to be the target genes of let-7e by luciferase reporter assays. We found that treatment with let-7e inhibitor could up-regulate the mRNA levels of all these genes (Additional file 2: Figure S1). These results confirmed the effectiveness of let-7e inhibitor. Then, we performed MTT assays to detect the corresponding alterations in cisplatin cytotoxicity. As shown in Fig. 2b, transfection of let-7e agomir decreased the IC_50_ of cisplatin from 6.60 μM to 3.66 μM in A2780 cells (*P* = 0.084), and from 20.05 μM to 9.66 μM in C13K cells (*P* = 0.012). Inversely, treatment with let-7e inhibitor increased the IC_50_ of ciaplatin in OV2008 cells from 5.77 μM to 9.46 μM (*P* = 0.042). The effect of let-7e on response to cisplatin was further confirmed in colony-formation assays (Fig. 2c–e). These results confirm that the low let-7e expression in EOC is associated with platinum-resistance.

### Let-7e leads to defect in repair of DNA double-strand break induced by cisplatin

**Fig. 3:**
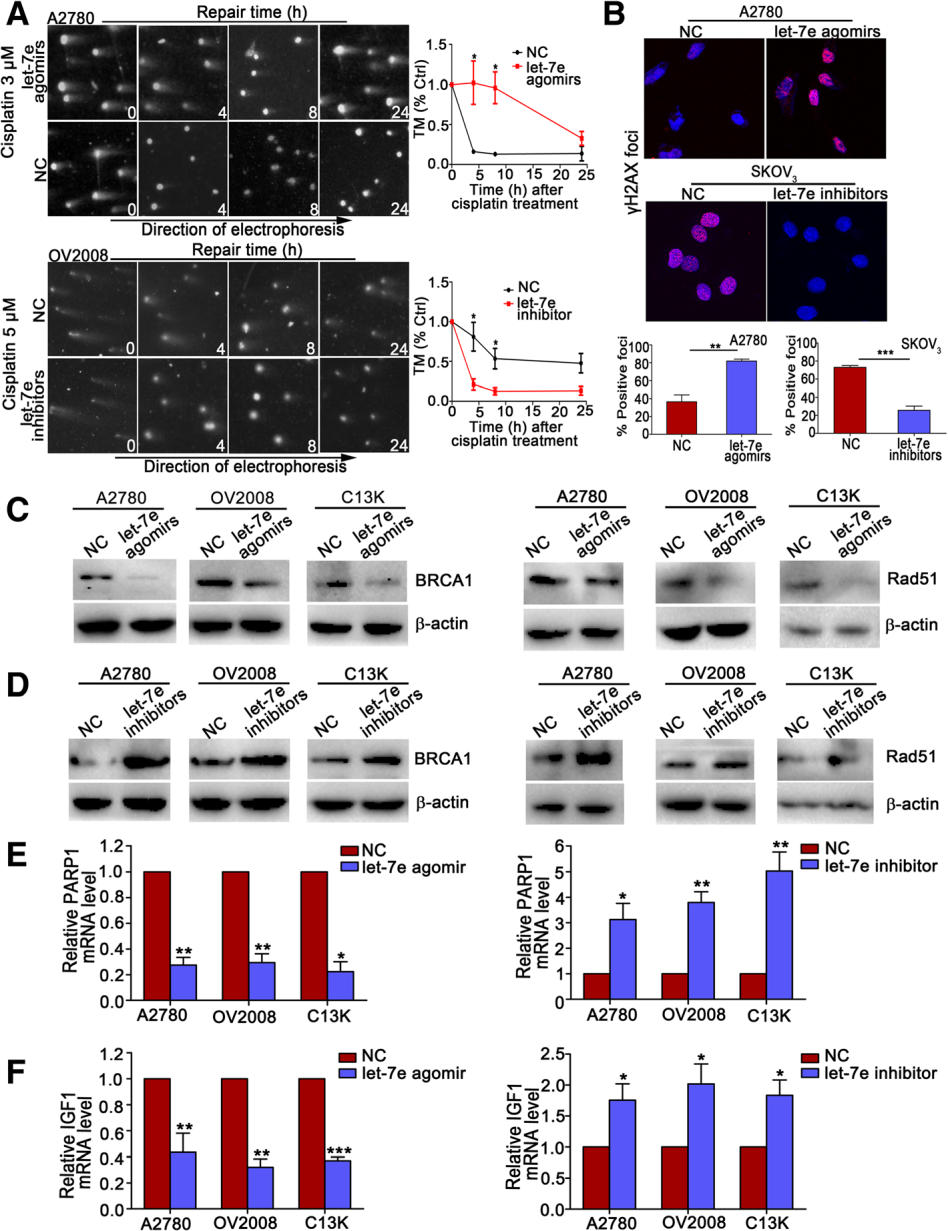
Overexpression of let-7e decreases the DSB repair capacity of EOC cell lines treated with cisplatin. **a** DSB repair dynamics of transfected A2780 and OV2008 cells after treatment with cisplatin measured by neutral comet assay. The TM values measured at 0 h was set as 100% and residual DNA damage at 4, 8, 24 h after cisplatin incubation was expressed as percent of that at 0 h. Representative images are shown on the left and the mean ± SD for each group in the right panel. **b** After treatment with cisplatin, γ-H2AX foci in A2780 transfected with let-7e agomirs and SKOV_3_ with let-7e inhibitors were examined 8 h after recovery. The percentage of positive cells (with five or more foci) were used for the comparison between groups. Representative images and the mean ± SD for each group are shown. **c** and **d** Western bolt shows the BRCA1 and Rad51 levels of cell lines transfected with let-7e agomirs or inhibitors. **e** and **f** qRT-PCR analyses show the mRNA levels of PARP1 and IGF1 in cell lines transected with let-7e agomirs or inhibitors. Student’s *t*-test, *P < 0.05, **P < 0.01, ***P < 0.001

Enhanced DNA DSB repair is an important mechanism of drug resistance. Having clarified the role of let-7e in cisplatin-resistance, we next wondered whether let-7e influences the repair capacity of DSB induced by cisplatin in EOC. We assessed the kinetics of DSB repair in EOC cells after exposure to cisplatin by using neutral single-cell electrophoresis. As shown in Fig. 3a, the DSB induced by cisplatin treatment began to recover 4 h after cisplatin stimulation in control A2780 cells, whereas increased let-7e expression led to a dramatically delayed DSB repair. Conversely, down-regulation of let-7e in OV2008 cells resulted in an accelerated DSB repair compared with the negative control. We next examined whether let-7e regulates the formation of γH2AX foci after incubation with cisplatin, which is a surrogate marker of DSB [15]. Significantly increased cells with positive γH2AX foci were observed in the A2780 cells transfected with let-7e agomirs compared with control, while a remarkable decrease in γH2AX foci was shown in the SKOV_3_ cells transfected with let-7e inhibitors compared with control (Fig. 3b and Additional file 2: Figure S2).

Given that Rad51 and BRCA1 play a central role in HR-mediated DSB repair [2, 24, 25] and govern the response of tumor cells to cisplatin [26, 27], we wondered whether let-7e regulates BRCA1 and Rad51 expression in EOC. Werstern blotting assays showed that the let-7e overexpression in A2780, OV2008 and C13K cells substantially decreased BRCA1 and Rad51 expression (Fig. 3c). Inversely, after transfection with let-7e inhibitors, BRCA1 and Rad51 protein levels were significantly increased compared with control (Fig. 3d). These findings suggest that let-7e inhibits the repair of cisplatin-induced DSB might be partially achieved by down-regulating BRCA1 and Rad51.

To further investigate the mechanisms of how let-7e impacts BRCA1 and Rad51 expression, we used miRWalk (http://zmf.umm.uni-heidelberg.de/apps/zmf/mirwalk2/) and microRNA.org (http://www.microrna.org/) to predict the target genes of let-7e. Among the search results, poly (ADP-ribose) polymerase 1 (PARP1) and insulin-like growth factor-1 (IGF-1) captured our attention because of their involvement in the regulation of BRCA1 and Rad51 expression and DSB repair [28, 29]. After treatment with let-7e agomir, the mRNA levels of PARP1 and IGF-1 were markedly decreased. Conversely, let-7e downregulation substantially increased the expression of PARP1 and IGF-1 (Fig. 3e and f).

### Expression and prognostic values of let-7e, BRCA1 and Rad51 in epithelia ovarian cancer

**Fig. 4:**
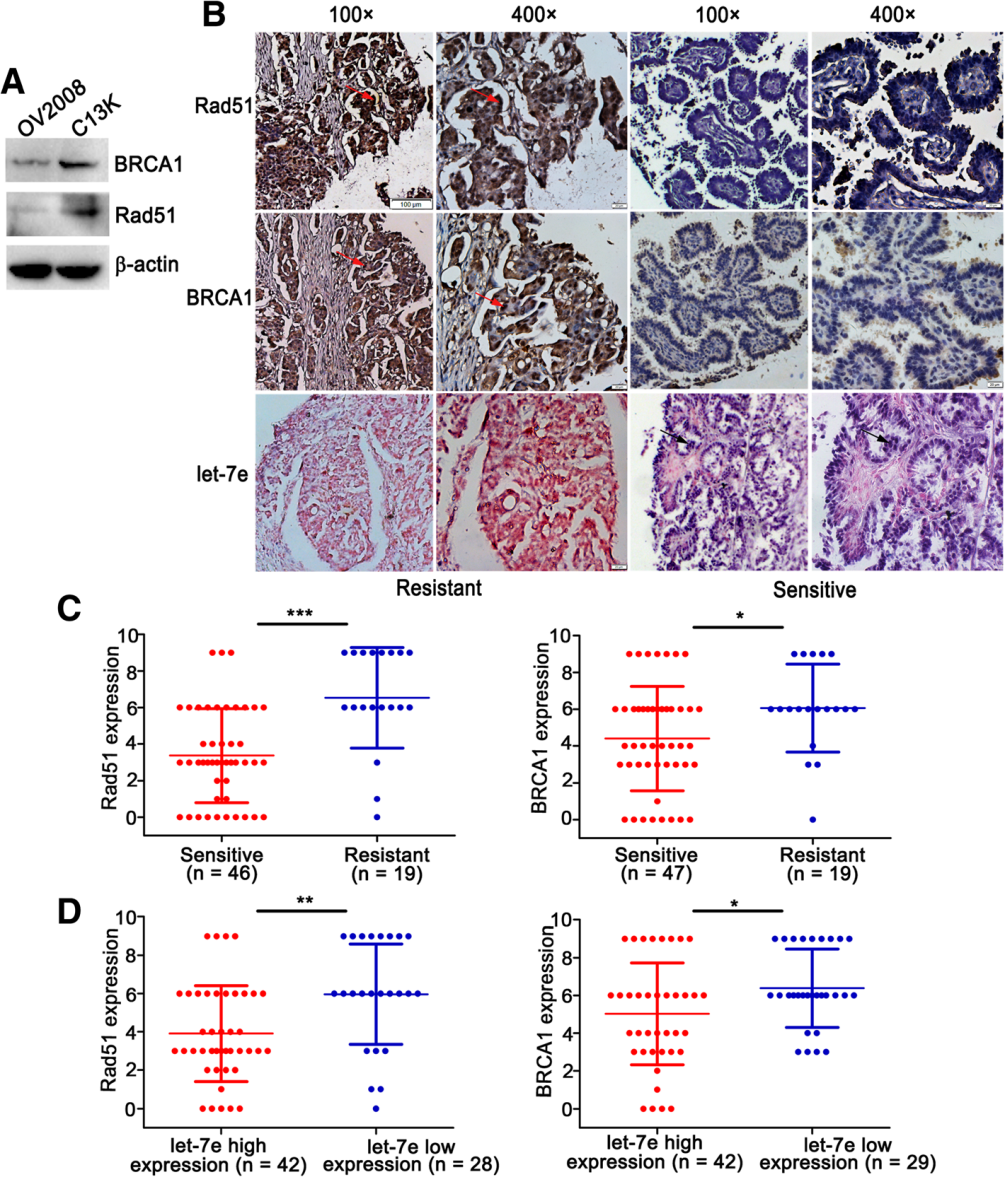
Expression of let-7e, BRCA1 and Rad51 in EOC. **a** Results of western bolt analysis of BRCA1 and Rad51 protein expression in cisplatin-sensitive and resistant cells. **b** Representative images of let-7e expression via ISH and BRCA1, Rad51 via IHC in one of the chemo-resistant or sensitive ovarian cancer tissues. The let-7e LNA-ISH signal is blue (black arrow) and IHC positive staining is yellow (red arrow). **c** Semi-quantification of BRCA1 and Rad51 expression in chemo-resistant versus chemo-sensitive EOC tissues. *P*_*BRCA1*_ = 0.0251, *P*_*Rad51*_ = 0.0001. **d** Expression of Rad51 and BRCA1 in let-7e high and low level cohort. *P*_*BRCA1*_ = 0.0383, *P*_*Rad51*_ = 0.0016. Mann Whitney test, *P < 0.05, **P < 0.01, ***P < 0.001

Given the inhibitory regulation of BRCA1 and Rad51 by let-7e in vitro, a negative relationship of let-7e with BRCA1 and Rad51 in EOC is expected. Contrary to let-7e expression, western blotting showed increased protein levels of BRCA1 and Rad51 in C13K cells compared with its paired sensitive line OV2008 (Fig. 4a). In line with the results in cells, IHC revealed significantly elevated expression of BRCA1 and Rad51 in the chemo-resistant EOC tissues compared with the chemo-sensitive cases (*P*_*BRCA1*_ = 0.0251, *P*_*Rad51*_ = 0.0001; Fig. 4b-c). In addition, both BRCA1 and Rad51 expression were negatively correlated with let-7e (*P*_*BRCA1*_ = 0.0383, *P*_*Rad51*_ = 0.0016; Fig. 4b and d).

**Fig. 5:**
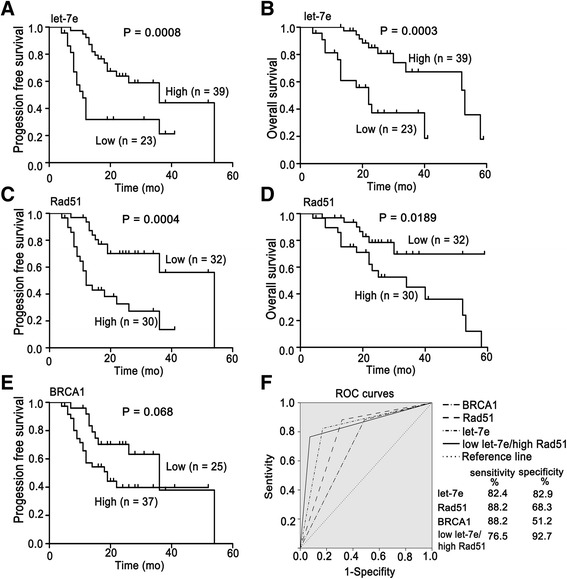
Influence of let-7e, BRCA1 and Rad51 on predicting survival and sensitivity to chemotherapy in EOC. **a–e** Kaplan-Meier survival curves for EOC patients based on the expression of let-7e, Rad51 and BRCA1. a, PFS for let-7e; b, OS for let-7e; c, PFS for Rad51; d, OS for Rad51; e, PFS for BRCA1. Ovarian cancer patients were stratified into let-7e, Rad51 or BRCA1 low and high expression groups according to the mean scores of their expression. Log-rank test. **f** ROC curves of let-7e, Rad51, BRCA1 and the combination of low let-7e and high Rad51 (*n* = 58). *AUC*_*let-7e*_ = 0.826, *P* < 0.0001; *AUC*_*Rad51*_ = 0.783, *P* = 0.001; *AUC*_*BRCA1*_ = 0.697, *P* = 0.019; *AUC*_*low let-7e/high Rad51*_ = 0.846, *P* < 0.0001

To investigate the possible prognostic values of let-7e, BRCA1 and Rad51 in ovarian cancer, we first analyzed their correlation with the clinicopathological characteristics of EOC patients. Low let-7e, high BRCA1 and high Rad51 were closely related with chemotherapy resistance (*P*_*let-7e*_ < 0.0001, *P*_*BRCA1*_ = 0.013, *P*_*Rad51*_ < 0.0001). Nevertheless, their expression was not significantly associated with age, FIGO stage, tumor categories or histological subtypes (Table 1). Next, univariate and multivariate Cox proportional hazards regression analyses were performed to assess the association between the expression of let-7e, BRCA1 and Rad51 and survival of patients. The univariate analyses indicated that advanced FIGO stage, low let-7e and high Rad51 were significantly associated with poor PFS and OS (Table 2). These results were confirmed by Kaplan-Meier analyses and log-rank tests (*P*_*PFS/let-7e*_ = 0.0008, *P*_*OS/let-7e*_ = 0.0003, *P*_*PFS/Rad51*_ = 0.0004, *P*_*OS/Rad51*_ = 0.0189, *P*_*PFS/BRCA1*_ = 0.068; Fig. 5a–e). The prognostic significance of BRCA1 and Rad51 was consistent with the observations in The Cancer Genome Atlas (TCGA) and Gene-Expression Omnibus database (Additional file 2: Figure S3). Multivariate analyses showed that low let-7e and high Rad51 were independent prognostic factors for OS (HR 3.235, 95% CI 1.258–8.317) and PFS (HR 2.519, 95% CI 1.098–5.779) in EOC, respectively (Table 3).

**Table 1:** Associations of let-7e, BRCA1 and Rad51 expression with clinicopathological characteristics of EOC patients

Clinicopathological Features	No. of Cases	Let-7e expression No.(%)			No. of Cases	BRCA1 expression No.(%)			Rad51 expression No.(%)		
		Low	High	*P* Value ^a^		Low	High	*P* Value ^a^	Low	High	*P* Value ^a^
Age at surgery, y											
<50	25	10 (40.0)	15 (60.0)	1	26	11 (42.3)	15 (57.7)	0.800	11 (42.3)	15 (57.7)	0.126
≥50	40	15 (37.5)	25 (62.5)		39	19 (48.7)	20 (51.3)		25 (64.1)	14 (35.9)	
Histological subtypes											
Serous	53	22 (41.5)	31 (58.5)	0.344	52	24 (46.2)	28 (53.8)	1	27 (51.9)	25 (48.1)	0.355
Others	12	3 (25.0)	9 (75.0)		13	6 (46.2)	7 (53.8)		9 (69.2)	4 (30.8)	
FIGO stage											
I–II	17	5 (29.4)	12 (70.6)	0.563	18	9 (50.0)	9 (50.0)	0.784	13 (72.2)	5 (27.8)	0.104
III–IV	48	20 (41.7)	28 (58.3)		47	21 (44.7)	26 (55.3)		23 (48.9)	24 (51.1)	
Tumor categories											
Type I	15	3 (20.0)	12 (80.0)	0.133	16	8 (50.0)	8 (50.0)	0.778	11 (68.7)	5 (31.3)	0.258
Type II	50	22 (44.0)	28 (56.0)		49	22 (44.9)	27 (55.1)		25 (51.0)	24 (49.0)	
Chemotherapy response											
Sensitive	43	8 (18.6)	35 (81.4)	<0.0001	46	26 (56.5)	20 (43.5)	0.013	33 (71.7)	13 (28.3)	<0.0001
Resistant	22	17 (77.3)	5 (22.7)		19	4 (21.1)	15 (78.9)		3 (15.8)	16 (84.2)	

*EOC* epithelial ovarian cancer, *FIGO* International Federation of Gynecology and Obstetrics

^a^ Fisher exact test

**Table 2:** Associations of different parameters with PFS, OS and chemotherapy response of EOC patients

		PFS ^a^		OS ^a^		Case No. (%)	Chemotherapy response ^b^	
Variables	Case No. (%)	HR (95% CI)	*P* Value	HR (95% CI)	*P* Value		OR (95% CI)	*P* Value
Age at surgery, y								
<50	25 (40.3)	0.921 (0.439–1.932)	0.828	0.763 (0.329–1.771)	0.529	23 (40.3)	1.094 (0.346–3.461)	0.879
≥50	37 (59.4)	1		1		35 (59.4)	1	
Histological subtypes								
Serous	50 (80.6)	1.493 (0.567–3.93)	0.417	1.153 (0.390–3.412)	0.796	46 (80.6)	2.419 (0.47–12.454)	0.291
Others	12 (19.4)	1		1		12 (19.4)	1	
FIGO stage								
I-II	18 (29.0)	0.352 (0.123–1.012)	0.053	0.143 (0.019–1.063)	0.057	16 (29.0)	0.257 (0.051–1.287)	0.098
III-IV	44 (71.0)	1		1		42 (71.0)	1	
Tumor categories								
Type I	15 (24.2)	0.647 (0.263–1.587)	0.341	0.762 (0.281–2.071)	0.595	15 (24.2)	0.518 (0.126–2.136)	0.363
Type II	47 (75.8)	1		1		43 (75.8)	1	
Let-7e expression								
Low	23 (37.1)	3.204 (1.541–6.661)	0.002	4.368 (1.804–10.575)	0.001	21 (37.1)	22.667 (5.114–100.457)	<0.0001
High	39 (62.9)	1		1		37 (62.9)	1	
BRCA1 expression								
Low	25 (40.3)	1	0.078	1	0.350	23 (40.3)	1	0.011
High	37 (59.7)	1.993 (0.925–4.294)		1.510 (0.636–3.585)		35 (59.7)	7.875 (1.594–38.906)	
Rad51 expression								
Low	32 (51.6)	1	0.001	1	0.025	30 (51.6)	1	0.001
High	30 (48.4)	3.584 (1.668–7.701)		2.752 (1.133–6.682)		28 (48.4)	16.154 (3.212–81.252)	

*EOC* epithelial ovarian cancer, *HR* hazards ratio, *CI* confidence interval, *PFS* progression-free survival, *OS* overall survival, *OR* odds ratio, *FIGO* International Federation of Gynecology and Obstetrics

^a^ Univariate Cox proportional hazards Regression analysis; ^b^ Univariate logistic regression analysis

**Table 3:** Multivariate regression analysis of significant prediction factors on PFS,OS and chemotherapy response of EOC patients

		PFS ^a^		OS ^a^			Chemotherapy response ^b^	
Variables	Case No. (%)	HR (95% CI)	*P* Value	HR (95% CI)	*P* Value	Case No. (%)	OR (95% CI)	*P* Value
FIGO stage								
I–II	18 (29.0)	0.552 (0.184–1.658)	0.290	0.204 (0.027–1.551)	0.125	16 (29.0)	-	-
III–IV	44 (71.0)	1		1		42 (71.0)		
Let-7e expression								
Low	23 (37.1)	2.037 (0.912–4.554)	0.083	3.235 (1.258–8.317)	0.015	21 (37.1)	19.696 (3.477–111.572)	0.001
High	39 (62.9)	1		1		37 (62.9)	1	
BRCA1 expression								
Low	25 (40.3)	-	-	-	-	23 (40.3)	1	0.155
High	37 (59.7)					35 (59.7)	5.042 (0.541–47.011)	
Rad51 expression								
Low	32 (51.6)	1	0.029	1	0.404	30 (51.6)	1	0.163
High	30 (48.4)	2.519 (1.098–5.779)		1.500 (0.579–3.891)		28 (48.4)	4.290 (0.553–33.26)	

*EOC* epithelial ovarian cancer, *HR* hazards ratio, *CI* confidence interval, *PFS* progression-free survival, *OS* overall survival, *OR* odds ratio, *FIGO* International Federation of Gynecology and Obstetrics

^a^ Multivariate Cox proportional hazards Regression analysis; ^b^ Multivariate logistic regression analysis

In order to evaluate the capacity of let-7e, BRCA1 and Rad51 expression to predict the sensitivity of EOC to platinum-taxane chemotherapy, ROC curves were established. The area under the curve (AUC) for let-7e, Rad51 and BRCA1 were 0.826 (sensitivity 82.4%, specificity 82.9%), 0.783 (sensitivity 88.2%, specificity 68.3%) and 0.697 (sensitivity 88.2%, specificity 51.2%) respectively (Fig. 5f). These results were further confirmed by univariate logistic regression analyses which showed that low let-7e, high Rad51 and high BRCA1 were all significantly correlated with chemotherapy resistance (Table 2). However, only let-7e was proved to be an independent factor to predict chemotherapy response indicated by multivariable logistic analysis (*P*_*let-7e*_ = 0.001; OR, 19.696; 95% CI, 3.477–111.572; Table 3). In addition, the AUC of the ROC curve for the combination of low let-7e and high Rad51 achieved 0.846 with a specificity of 92.7% and a sensitivity of 76.5%.

## Discussion

Among the let-7 family members, let-7a and let-7b are proposed to be important regulators of chemo-response in tumor through regulating chemotherapeutic agents-induced apoptosis [10, 30]. Our previous study showed that let-7e was down-regulated in cisplatin-resistant EOC cell lines and re-expression of let-7e could sensitize cancer cells to cisplatin in vitro and in vivo. In the present study, we demonstrated that let-7e determines the fate of the EOC cells exposed to cisplatin through governing the repair of cisplatin-induced DSB and further verified the association between loss of let-7e and cisplatin-resistance in human EOC tissues. In addition, low let-7e was found to be an independent prediction factor for poor survival and resistance to platinum-based chemotherapy. These findings suggest that let-7e, similar to let-7a and let-7b, plays an essential role in cisplatin-resistance and might be a promising predictor for chemotherapy response in EOC.

To survive and maintain genome integrity after receiving exogenous or endogenous stimuli, cells initiate their cellular DNA damage response system. DSB is the most deleterious form of DNA lesions, which leads to cell death when DNA repair was faulty or insufficient. BRCA1 and Rad51 are essential for HR-mediated DSB repair and are identified as targets of several miRNAs. MiR-9 inhibited cisplatin-induced BRCA1 and Rad51 foci formation by directly targeting BRCA1, leading to impaired DNA damage repair and restored sensitivity to cisplatin [31]. MiR-146a, miR-146b-5p and miR-182 are also reported to bind to the 3’-UTR of BRCA1, resulting in a reduction in BRCA1 and HR [32]. Over-expression of miR-506 and miR-103/107 decreased Rad51 expression and subsequently reduced HR-directed repair of DNA damage induced by cisplatin, leading to an augmented response to cisplatin [33, 34]. Here, we demonstrated that enhanced let-7e could reduce the repair of cisplatin-induced DSB and re-sensitize EOC cells to cisplatin through down-regulating BRCA1 and Rad51.

As we known, miRNA regulates gene expression through binding to the 3’-UTR of targeting mRNAs based on base paring. However, the 3’-UTRs of BRCA1 and Rad51 contain no putative let-7e binding site, suggesting that let-7e cannot directly target BRCA1 or Rad51 mRNA. Hegan DC et al*.* found that inhibition of PARP1 suppressed BRCA1 and Rad51 expression through stimulating repressive E2F4/p130 complexes to bind to the promoters of BRCA1 and Rad51 [28]. Jeon JH et al*.* reported that IGF-1 attenuated the DNA repair of cisplatin-induced DSB, γ-H2AX foci formation and damage checkpoint pathway in non-small cell lung cancer [29]. Both PARP1 and IGF-1 are potential direct targets of let-7e according to miRWalk and microRNA.org, our study showed that let-7e negatively regulated the expression of PARP1 and IGF-1 in ovarian cancer cell lines, indicating that PARP1 and IGF-1 might link let-7e to Rad51 and BRCA1, although that remains to be verified in further studies.

The expression and function of let-7e are varied in different tumors. Let-7e was down-regulated in colorectal cancer, non-small-cell lung carcinoma, esophageal cancer, lymphoma and ovarian cancer and functioned as a tumor suppressor [35–39]. In contrast, an increased let-7e expression was observed in retinoblastoma and synovial sarcoma [40, 41]. These results suggest that the role of let-7e is tumor-specific. Although low let-7e was observed in ovarian cancer, its role in oncogenesis and progression of EOC remains unclear. In the present study, we found let-7e was decreased in chemo-resistant ovarian cancers compared with chemo-sensitive cases and was identified as an independent predictor for poor survival and chemo-resistance, suggesting a tumor-suppressive role of let-7e in EOC and highlighting the clinical value of let-7e for stratification of patients with risk to develop chemo-resistance and to relapse. Moreover, let-7e emerged as a potential circulating biomarker for diseases. Serum let-7e level was significantly correlated with tumor size and metastasis status in papillary thyroid carcinoma [42]. Low serum let-7e was identified as a potential predictor for severe knee or hip osteoarthritis [43]. The plasma let-7e was found to be an early marker of metabolic syndrome in pediatric population [44]. Thus, future investigations should strive to incorporate let-7e detection, both in EOC tissue and in peripheral circulation, into clinical trials to evaluate whether let-7e can be used to guide decision making on usage of platinum-taxane chemotherapy and monitoring the response to chemotherapy during treatment in EOC.

Also, let-7e has been implicated in taxane resistance. The expression of almost all the let-7 family members (let-7a, let-7c, let-7d, let-7e, let-7f, let-7 g, let-7i and mir-98) was reduced in a paclitaxel-resistant hepatocellular carcinoma cell line [45]. Ectopic let-7a, let-7b, let-7c and let-7 g expression rendered tumor cells more sensitive to taxol treatment in breast, pancreatic, colorectal, lung and ovarian cancer [46–49]. Although less frequent, upregulation of certain let-7 family members has also been observed in taxane resistant cancer cells. Tsang et al. demonstrated that enforced let-7a induced the resistance to apoptosis caused by paclitaxel in squamous carcinoma A431 cells and hepatocellular carcinoma HepG2 cells [50]. In ovarian cancer, let-7e was upregulated in paclitaxel resistant A2780 cells [51]. These conflicting data indicate that the function of let-7 family in taxane resistance may be tissue or cell type specific. Specially, contrast to the upregulation of let-7e in paclitaxel resistant EOC cell, let-7e was down-regulated in cisplatin-resistant cell lines, suggesting that the role of let-7e in chemo-resistance may be drug-specific.

## Conclusions

Our data demonstrate that low let-7e expression contributes to the development of chemo-resistance in EOC through impairing DNA DSB repair via regulating the expression of BRCA1 and Rad51 and is a potential predictor for poor survival and resistance to platinum-taxane chemotherapy in patients with EOC. However, the mechanism through which let-7e regulates BRCA1 and Rad51 remains unknown and the prognostic value of let-7e needs to be validated in prospective, large-scale studies.

## Additional files

Additional file 1: Table S1. The sequences of PCR primers used in this study. (DOCX 13 kb)

Additional file 2: Figure S1. Influence of let-7e inhibitor on the mRNA levels of RFX6, CASP3, MMP9 and EZH2. Student’s *t*-test, *P < 0.05, **P < 0.01, ***P < 0.001. **Figure S2.** qRT-PCR analysis of let-7e expression in SKOV_3_ cells after transfection with let-7e inhibitor. Student’s t-test, *** *P* < 0.001. **Figure S3.** Associations of Rad51 and BRCA1 expression with prognosis of ovarian cancer patients in publically available datasets. (DOCX 202 kb)

## Abbreviations

AP: Alkaline phosphatase
ATM: Ataxia telangiectasia mutated
ATR: Ataxia Rad3-related
AUC: Area under the curve
BSA: Bovine serum albumin
CASP: Comet Assay Software Project
CASP3: Caspase 3
CCTCC: China Center for Type Culture Collection
CI: Confidence interval
DMEM: Dulbecco’s Modified Eagle Medium
DSB: Double strand break
EOC: Epithelial ovarian cancer
EZH2: Enhancer of zeste 2
FFPE: Formalin-fixed and paraffin embedded
FIGO: International Federation of Gynecology and Obstetrics
HR: Hazards ratio
HR: Homologous recombination
IGF-1: insulin-like growth factor-1
IHC: Immunohistochemistry
IR: Ionizing radiation
ISH: In situ hybridization
LMP: Low melting point
LNA: Locked Nucleic Acids
MMP9: Matrix metalloproteinase-9
NHEJ: Non-homologous end-joining
OS: Overall survival
PARP1: Poly(ADP-ribose) polymerase 1
PBST: PBS containing 0.1% Tween-20
PFS: Profession-free survival
RFX6: Regulatory factor X 6
ROC: Receiver operating characteristic
SSC: Standard saline citrate
TM: Tail moment
UTR: Untranslated region
