# Supplementary material for: Let-7e sensitizes epithelial ovarian cancer to cisplatin through repressing DNA double strand break repair
Source: J Ovarian Res. 2017 Apr 4;10:24. doi: 10.1186/s13048-017-0321-8 (PMC5379542; doi:10.1186/s13048-017-0321-8)
Supplement: Supplementary file 1 — The sequences of PCR primers used in this study. (DOCX 13 kb) [file 13048_2017_321_MOESM1_ESM.docx]

**Additional file 1**

**Table S1.** The sequences of PCR primers used in this study.

| Genes | Primers | |
| --- | --- | --- |
|  | Upstream | Downstream |
| RFX6 | GCAAGCCGAGGAAGTGTC | GAGGGAGAGTGGGATAAATGG |
| CASP3 | AGAACTGGACTGTGGCATTGAG | GCTTGTCGGCATACTGTTTCAG |
| MMP9 | CATTCAGGGAGACGCCCA | AACCACGACGCCCTTGC |
| EZH2 | TTGTTGGCGGAAGCGTGTAA AATC | TCCCTAGTCCCGCGCAATGA GC |
| PARP1 | CGAGTATTACTATTAGCCCTTGGG | AACATGGGAGCTCTTGAAATATG |
| IGF1 | CACTTCTTTCTACACAACTCGGGC | CGACTTGCTGCTGCTTTTGAG |
| β-actin | CAGAGCCTCGCCTTTGCC | GTCGCCCACATAGGAATC |
