# Supplementary material for: Let-7e sensitizes epithelial ovarian cancer to cisplatin through repressing DNA double strand break repair
Source: J Ovarian Res. 2017 Apr 4;10:24. doi: 10.1186/s13048-017-0321-8 (PMC5379542; doi:10.1186/s13048-017-0321-8)
Supplement: Supplementary file 2 — Influence of let-7e inhibitor on the mRNA levels of RFX6, CASP3, MMP9 and EZH2. Student’s t-test, *P < 0.05, **P < 0.01, ***P < 0.001. Figure S2. qRT-PCR analysis of let-7e expression in SKOV3 cells after transfection with let-7e inhibitor. Student’s t-test, *** P < 0.001. Figure S3. Associations of Rad51 and BRCA1 expression with prognosis of ovarian cancer patients in publically available datasets. (DOCX 202 kb) [file 13048_2017_321_MOESM2_ESM.docx]

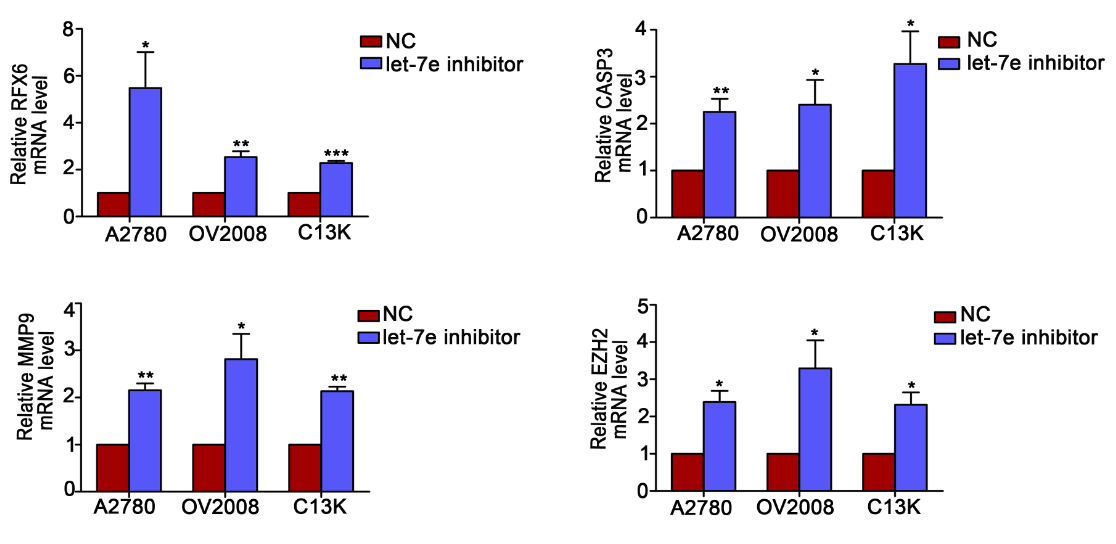


**Figure S1.** Influence of let-7e inhibitor on the mRNA levels of RFX6, CASP3, MMP9 and EZH2. Student’s t-test, **P* < 0.05, ***P* < 0.01, ****P* < 0.001


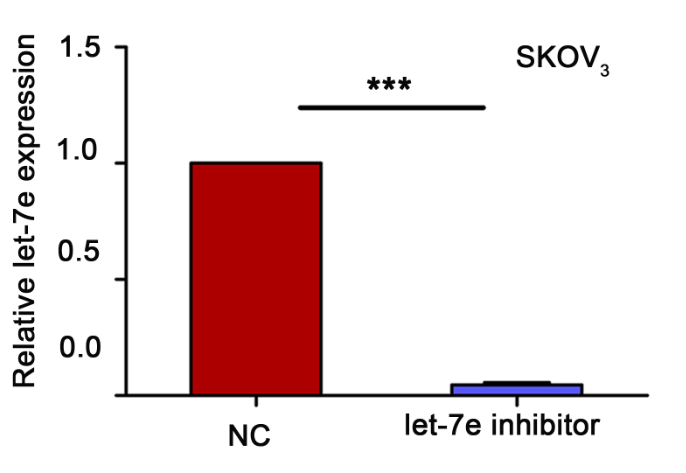


**Figure S2.** QRT-PCR analysis of let-7e expression in SKOV3 cells after transfection with let-7e inhibitor. Student’s t-test, *** *P* < 0.001


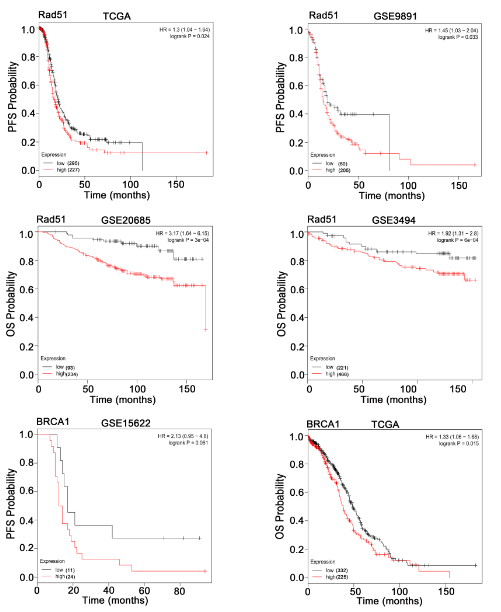


**Figure S3.** Associations of Rad51 and BRCA1 expression with prognosis of ovarian cancer patients in publically available datasets.
